# Oxaliplatin‐induced portal hypertension manifesting as portosystemic shunt: A case report and review of sinusoidal obstruction syndrome in gastric cancer

**DOI:** 10.1002/deo2.70109

**Published:** 2025-03-29

**Authors:** Toshimitsu Ichimaru, Takamitsu Sakamoto, Teruyoshi Amagai

**Affiliations:** ^1^ Department of Gastroenterology Fukuoka Tokushukai General Hospital Fukuoka Japan; ^2^ Department of Clinical Engineering Faculty of Health Care Sciences Jikei University of Health Care Sciences Osaka Japan

**Keywords:** advanced gastric cancer, oxaliplatin, portal hypertension, portosystemic shunt, sinusoidal obstructive syndrome

## Abstract

A 77‐year‐old woman with stage IVB gastric adenocarcinoma (cT4N2M1) received 10 cycles of S‐1, oxaliplatin, and nivolumab combination chemotherapy. Imaging and biopsy confirmed a complete response. However, thrombocytopenia, mild splenomegaly, and elevated liver enzymes developed following oxaliplatin administration. After the tenth cycle, a contrast‐enhanced computed tomography scan revealed a portosystemic shunt. Oxaliplatin was discontinued, but the shunt persisted after 1 year. Balloon‐occluded retrograde transvenous obliteration was successfully performed, resulting in the resolution of the shunt. This case highlights the potential for oxaliplatin‐induced portal hypertension requiring close monitoring.

## INTRODUCTION

Gastric cancer remains a significant clinical challenge, with some cases diagnosed at an advanced stage. Oxaliplatin, a third‐generation platinum‐based chemotherapeutic agent, is frequently used in combination with S‐1 and nivolumab for unresectable gastric cancer. Sinusoidal obstruction syndrome (SOS), a known adverse effect of oxaliplatin, can manifest as portal hypertension. This case report describes a patient with oxaliplatin‐associated SOS presenting with a portosystemic shunt successfully treated with prophylactic balloon‐occluded retrograde transvenous obliteration (BRTO).

### Case presentation

**FIGURE 1:**
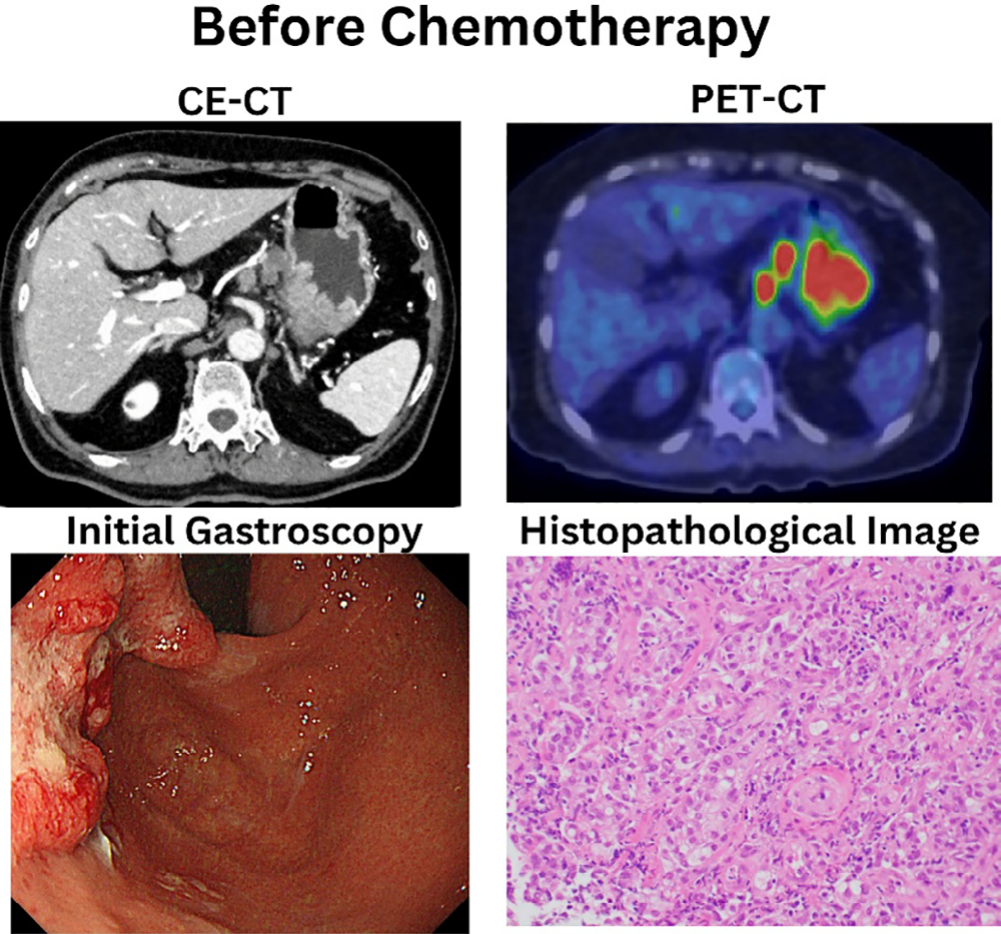
Positron emission tomography‐computed tomography (PET‐CT) shows abnormal accumulation in gastric artery lymph nodes and periaortic lymph nodes. Stomach biopsy shows adenocarcinoma (Group 5), HER2 negative. Gene testing (FISH) was negative. CE‐CT, contrast‐enhanced computed tomography.

A 77‐year‐old woman presented to Fukuoka Tokushukai General Hospital with advanced gastric cancer. Endoscopy revealed a neoplasm in the posterior wall of the upper gastric body and fundus, confirmed histologically as HER2‐negative adenocarcinoma (Figure 1). Contrast‐enhanced computed tomography scan (CE‐CT) and positron emission tomography (PET)‐CT staging revealed stage IVB disease (T4N3M1; Figure 1). She received 10 cycles of S‐1, oxaliplatin (SOX), and nivolumab for 8 months.

**FIGURE 2:**
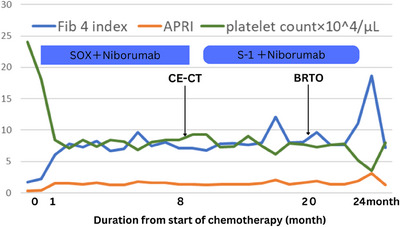
Course of treatment and changes in platelet count, Fibrosis‐4 index, and APRI. Abbreviations: APRI, aspartate aminotransferase to platelet ratio index; BRTO, balloon‐occluded transfemoral obliteration; CE‐CT, contrast medium‐enhanced computed tomography.

**FIGURE 3:**
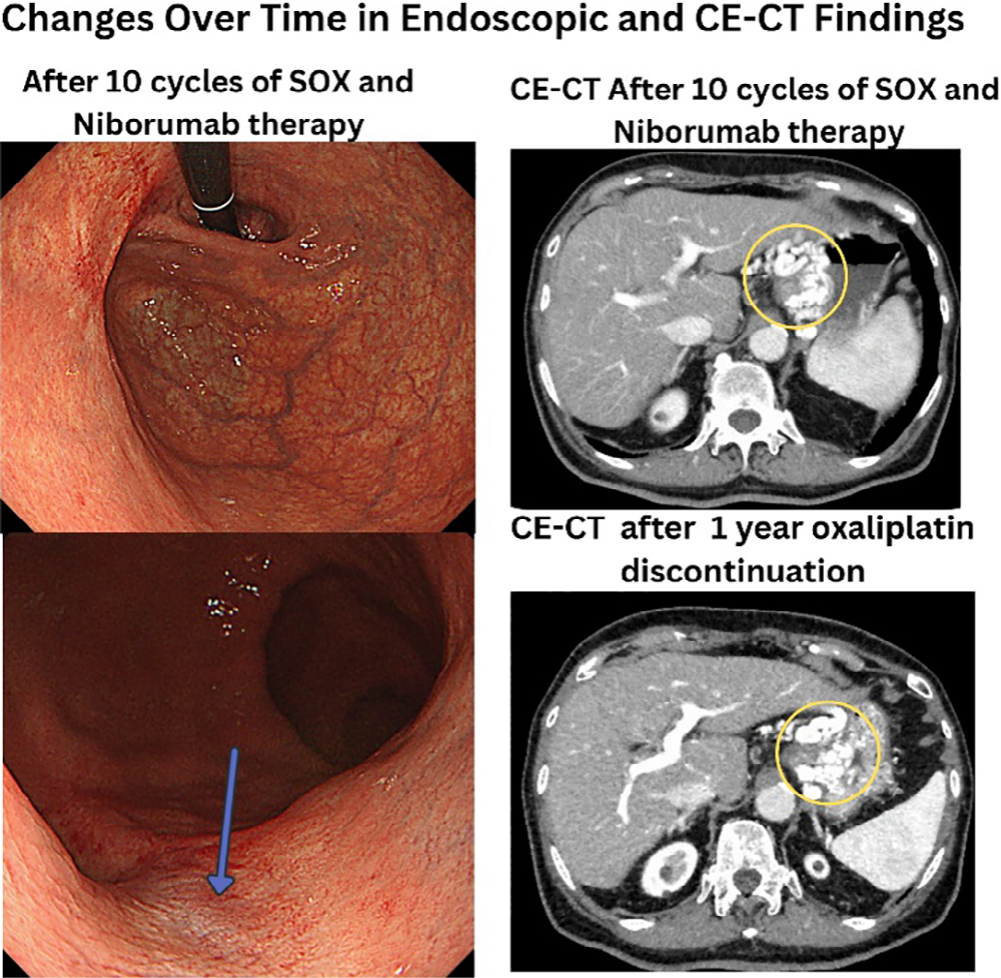
Endoscopic findings suspicious for the variceal‐like vessel (arrow), and complete response to chemotherapy. Contrast medium‐enhanced computed tomography (CE‐CT) shows a complete response to chemotherapy and the formation of a portosystemic shunt (circle) and suspected mild enlargement of the shunt 1 year after oxaliplatin discontinuation.

During SOX therapy, thrombocytopenia developed (Figure 2). Post‐chemotherapy CE‐CT demonstrated a complete response. However, subsequent gastroscopy revealed a complete response to chemotherapy, but also the presence of a variceal‐like vessel, confirmed by CE‐CT (Figure 3), which showed mild splenomegaly and enlargement of the portosystemic shunt. Oxaliplatin was discontinued after 8 months.

While the incidence of varices rupture is relatively low, it remains a clinically significant concern.

**FIGURE 4:**
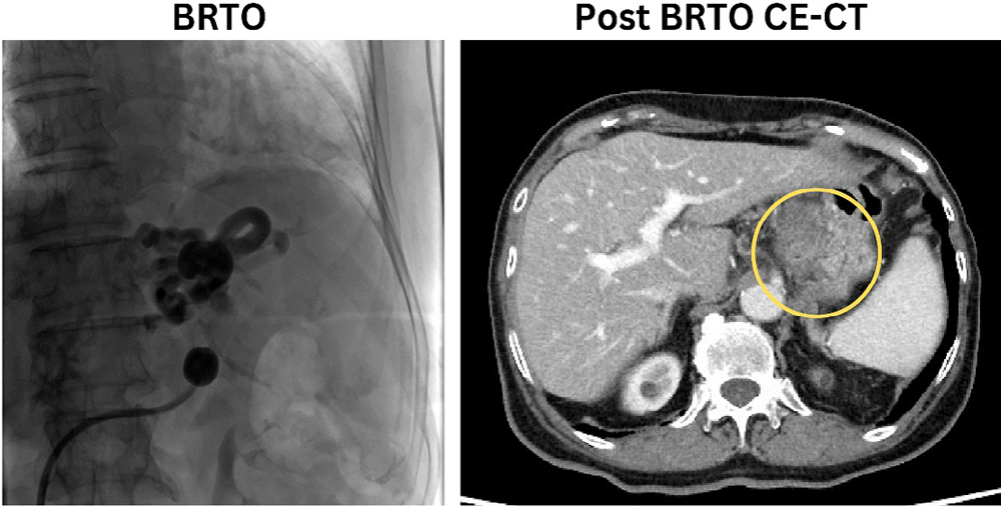
The venogram of balloon‐occluded transfemoral obliteration (BRTO) procedure. Post‐BRTO contrast‐enhanced computed tomography (CE‐CT) reveals occlusion of the portosystemic shunt (circle).

Although asymptomatic, she presented with hyperammonemia at 84 (reference range: 12–66 µg/dL). Persistent thrombocytopenia, enlargement of portosystemic shunt, and hyperammonemia prompted prophylactic BRTO (Figure 4) after a duration of 20 months from the initiation of chemotherapy. The postoperative course was uneventful, and no adverse event occurred, S‐1 and nivolumab therapy continued for 2 years. Postoperative ammonia data improved to 37 µg/dL, but platelet counts did not increase.

## DISCUSSION

This case report describes a 77‐year‐old woman with stage IVB gastric adenocarcinoma who developed portal hypertension after only 10 cycles of oxaliplatin‐based chemotherapy (SOX and nivolumab), a significantly shorter timeframe than the previously reported median of 50.4 months.^1^ This rapid onset of portosystemic shunt, coupled with their persistence even after oxaliplatin discontinuation, suggests a potentially aggressive form of oxaliplatin‐induced portal hypertension and irreversible damage. Furthermore, this case contributes to the limited literature on oxaliplatin‐induced portosystemic shunt, specifically in gastric cancer patients, highlighting a critical area needing further investigation. The successful resolution of the shunt using BRTO adds to the clinical experience with this intervention in this context. Finally, the case underscores the inadequacy of current diagnostic criteria for chemotherapy‐induced SOS, particularly in gastric cancer, emphasizing the need for refined diagnostic tools and a greater understanding of the long‐term consequences of oxaliplatin‐induced portal hypertension. To summarize SOS symptomatology, serology, and images, painful hepatomegaly, ascites, weight gain (>5% of pre‐chemotherapy), serum bilirubin (>2 mg/dL), transaminases (>2 times normal), and no specific findings in CT and MRI.^2^

Oxaliplatin, a cornerstone of first‐line chemotherapy regimens for advanced gastric cancer chemotherapy, is associated with various adverse effects, including neurotoxicity, gastrointestinal disturbances, and hematologic toxicities. However, a potentially serious and often under‐recognized long‐term complication is SOS, which can manifest as non‐cirrhotic portal hypertension.^3^ The incidence of SOS following oxaliplatin‐based chemotherapy ranges widely, from 19% to 52%,^4^ and its clinical presentation can be variable.

While oxaliplatin‐induced hepatic sinusoidal injury is well‐documented,^5^ the precise pathological mechanisms linking oxaliplatin exposure to non‐cirrhotic portal hypertension remain incomplete.^6^ While some studies report a median time to varices manifestation of 50.4 months after oxaliplatin initiation,^1^ our patient exhibited a more rapid onset of thrombocytopenia and portosystemic shunt, developing within 10 cycles of SOX chemotherapy.

The patient's presentation—thrombocytopenia, mild splenomegaly, and portosystemic shunt developing temporally after oxaliplatin administration—strongly suggests oxaliplatin‐induced SOS as the underlying etiology. The absence of other known risk factors for portal hypertension further supports this causal link. Although elevated liver enzymes are sometimes associated with SOS, the presence of portal hypertension, manifested by splenomegaly and varices, is a more reliable diagnostic indicator than isolated mild elevations in liver function tests. Furthermore, elevated aminotransferase to platelet ratio index (and Fibrosis‐4 scores, also associated with splenomegaly, can provide additional diagnostic support.^3^

Importantly, in our patient, portal hypertension appeared irreversible, with persistent thrombocytopenia and varices even after oxaliplatin discontinuation. This observation aligns with previous reports indicating that oxaliplatin‐induced SOS can lead to persistent portal hypertension. The commonly used Jones criteria for SOS diagnosis are primarily applicable to post‐hematopoietic stem cell transplantation settings^7^ and may not accurately reflect chemotherapy‐induced SOS. This highlights the need for the development of specific diagnostic criteria for chemotherapy‐related SOS, particularly in the context of gastric cancer, given the paucity of data in this specific patient population.^3^

This case underscores the critical importance of close monitoring for clinical signs of portal hypertension, including platelet counts, splenomegaly, aminotransferase to platelet ratio index, Fibrosis‐4, and endoscopic findings, in patients receiving oxaliplatin‐based chemotherapy for gastric cancer.^3^ Early detection allows for timely prophylactic interventions, such as BRTO in this instance, potentially preventing serious complications.^8^ The successful BRTO procedure in our patient further emphasizes the value of proactive management. Follow‐up is also necessary after BRTO with adequate concern for rupture hemorrhage of esophagogastric varices.

However, this case report, while informative, is limited by its inherent nature as a single case study.

Larger prospective studies with comprehensive pathological correlation are crucial to definitively establish the causal relationship between oxaliplatin and the development of gastric varices in gastric cancer patients and to refine diagnostic criteria for chemotherapy‐induced SOS. This case highlights the need for increased clinician awareness of this rare but potentially serious complication of oxaliplatin therapy in gastric cancer.

## CONFLICT OF INTEREST STATEMENT

None.

## ETHICS STATEMENT

This case report did not include any analysis of human or animal subjects by any of the authors.

## PATIENT CONSENT STATEMENT

The patient provided informed consent for this case report.

## References

[1] Huang X , Li F , Wang L *et al*. Endoscopic treatment of gastroesophageal variceal bleeding after oxaliplatin‐based chemotherapy in patients with colorectal cancer. Endoscopy 2020; 52: 727–35. 32380558 10.1055/a-1157-8611

[2] Mohty M , Malard F , Alaskar AS *et al*. Diagnosis and severity criteria for sinusoidal obstruction syndrome/veno‐occlusive disease in adult patients: A refined classification from the European Society for Blood and Marrow Transplantation (EBMT). Bone Marrow Transplant 2023; 58: 749–54. 37095231 10.1038/s41409-023-01992-8

[3] Ye J , Xie Y , Xu Y , Chen N , Tu Y . Case report: Oxaliplatin‐induced idiopathic non‐cirrhotic portal hypertension: A case report and literature review. Front Med 2023; 10: 1285064. 10.3389/fmed.2023.1285064 PMC10713788 38089870

[4] Zhang X , Gao YY , Song DZ , Qian BX . Isolated gastric variceal bleeding related to non‐cirrhotic portal hypertension following oxaliplatin‐based chemotherapy: A case report. World J Gastroenterol 2022; 28: 3524–31. 36158260 10.3748/wjg.v28.i27.3524 PMC9346464

[5] Slade JH , Alattar ML , Fogelman DR *et al*. Portal hypertension associated with oxaliplatin administration: Clinical manifestations of hepatic sinusoidal injury. Clin Colorectal Cancer 2009; 8: 225–30. 19822514 10.3816/CCC.2009.n.038

[6] Nagahama S , Ueno M , Terada K *et al*. Oxaliplatin‐induced sinusoidal obstructive syndrome: A comparative study on clinical and pathological findings. Kanzo 2022; 63: 372–80.

[7] Cairo MS , Cooke KR , Lazarus HM , Chao N . Modified diagnostic criteria, grading classification and newly elucidated pathophysiology of hepatic SOS/VOD after haematopoietic cell transplantation. Br J Haematol 2020; 190: 822–36. 32133623 10.1111/bjh.16557 PMC7483983

[8] Ogata H , Gushima T , Maruoka S *et al.* [A case of portal hypertension after 5‐fluorouracil, leucovorin, and oxaliplatin (mFOLFOX6) chemotherapy]. Jpn J Gastroenterol 2013; 110: 2119–26. 24305101

